# Rapid acquisition of novel written word-forms: ERP evidence

**DOI:** 10.1186/s12993-020-00173-7

**Published:** 2020-12-02

**Authors:** Beatriz Bermúdez-Margaretto, Yury Shtyrov, David Beltrán, Fernando Cuetos, Alberto Domínguez

**Affiliations:** 1 Centre for Cognition and Decision Making, Institute for Cognitive Neuroscience, National Research University Higher School of Economics, Moscow, Russian Federation; 2 Center of Functionally Integrative Neuroscience, Aarhus University, Aarhus, Denmark; 3 Instituto Universitario de Neurociencia (IUNE) and Facultad de Psicología, Universidad de La Laguna, Tenerife, Spain; 4 Facultad de Psicología, Universidad de Oviedo, Oviedo, Spain

**Keywords:** Word learning, ERP methodology, Cluster-based random permutation analysis, N400, P200

## Abstract

**Background:**

Novel word acquisition is generally believed to be a rapid process, essential for ensuring a flexible and efficient communication system; at least in spoken language, learners are able to construct memory traces for new linguistic stimuli after just a few exposures. However, such rapid word learning has not been systematically found in visual domain, with different confounding factors obscuring the orthographic learning of novel words. This study explored the changes in human brain activity occurring online, during a brief training with novel written word-forms using a silent reading task

**Results:**

Single-trial, cluster-based random permutation analysis revealed that training caused an extremely fast (after just one repetition) and stable facilitation in novel word processing, reflected in the modulation of P200 and N400 components, possibly indicating rapid dynamics at early and late stages of the lexical processing. Furthermore, neural source estimation of these effects revealed the recruitment of brain areas involved in orthographic and lexico-semantic processing, respectively.

**Conclusions:**

These results suggest the formation of neural memory traces for novel written word-forms after a minimal exposure to them even in the absence of a semantic reference, resembling the rapid learning processes known to occur in spoken language.

## Background

Human brain possesses an impressive ability to learn novel vocabulary, not only during the first years of life when language development is taking off but also in adulthood, when learning a foreign language or acquiring new terms in the native one. Moreover, this capability of learning new vocabulary is highly efficient, as the acquisition and representation of novel words^1^ unfolds in a particularly fast and accurate fashion. Thus, in spoken language domain, extensive behavioral research has consistently proven the acquisition of new vocabulary as a very fast process, with learning outcomes obtained after relatively short training periods, in some cases involving just a few exposures [22, 26, 27, 33, 42, 44, 45, 51, 66, 105]. Indeed, this process was referred to as *fast mapping* in early developmental studies, in which children showed rapid and incidental learning for the association between new auditory forms and their referents see [19, 20]. There is accumulating body of evidence from studies using methodologies such as fMRI [16, 92], PET [81] or EEG, [5, 53, 54, 102, 104, 115], suggesting the existence of a neural mechanism supporting the rapid learning of novel spoken words, whose activity can be traced by measuring brain signals before and after a learning session, or even online, during the process of learning. In particular, a number of recent ERP studies have reported an increase of brain activity in core language circuits (most crucially left temporal lobe) elicited by novel spoken word-forms after only a few minutes of auditory exposure to them, and even in the absence of meaning referent or active rehearsal [53, 54, 102, 104, 115]. Most typically, this modulation of brain activity is manifested rather early in the electrophysiological response, at ~50–150 ms after the novel spoken word-form’s disambiguation point (the point in time when the phonotactical configuration of specific stimulus diverges from other spoken word-forms with identical initial phonemes, enabling its identification as a unique item during speech perception). Crucially, this response enhancement, which is explained as an activation of a newly built lexical representation, has been found under both attentive [53, 54] and passive listening conditions [102, 104, 115], showing the formation of neural memory traces to be not only a very fast but to a large degree also an automatic process (see [103], for a review).

However, language is not confined to the auditory modality; visually presented language and the neural mechanisms underpinning the acquisition of novel vocabulary through reading are no less important. In this sense, the acquisition of new orthographic forms is considered crucial for reading fluency, as it allows a reader to transfer from a serial grapheme-to-phoneme decoding of the novel word to a holistic whole-word recognition strategy [100, 101]. A number of behavioral studies have reported effects suggestive of fast orthographic learning, which can be achieved by training novel word-forms in reading-aloud tasks involving very few (from four to six) repetitions [, 15, 61, 62, 68, 96, 109]. For instance, it has been found that such training significantly improves the speed and accuracy of novel word-form recognition, leading to the elimination of the so-called lexicality effect, i.e. the differences between novel and previously known words [96]. Such a short exposure to novel written words has been reported to reduce the naming latency difference between short and long novel words caused by the serial, letter-by-letter decoding of unfamiliar stimuli (the so-called length effect, [2, 61, 62, 68]). These findings clearly indicate the formation of directly-accessed orthographic representations in the mental lexicon, causing a change in the reading strategy for the trained words, evolving from serial decoding to a parallel, whole-form recognition strategy. Furthermore, similar to the spoken domain, several ERP studies have also provided evidence suggesting the existence of a neurophysiological mechanism which enables rapid formation of mental representations for novel written word-forms perceived visually [8, 14, 76, 82]. Most typically, these studies report the modulation of the N400, an ERP component considered to reflect the lexico-semantic processing of stimuli [55]. Thus, the rapid N400 decrease obtained in these studies, after only a handful of exposures to novel word-forms presented within meaningful contexts [8, 76, 82] or even after just one single visual presentation within a highly-constrained semantic context [14], is considered to reflect the facilitation in the processing of these stimuli and their integration in the lexico-semantic system through meaningful associations.

Therefore, the build-up process of new linguistic representations can be hypothesized to be a very fast process, both in spoken and visual domains. Some other studies suggest, however, that it is only after an intensive and meaningful training with novel words, involving a higher number of exposures (including even weekly training sessions) and consolidation periods (at least overnight, but often including days of practice), that it may be possible to ensure the build-up of new representations fully-integrated into the mental lexicon, both in spoken [27, 33, 42, 72] and in visual modalities [6, 17, 73, 107]. Although apparently contradictory at first glance, both sets of findings—those supporting rapid fast mapping and those favoring slower learning—likely reflect two different stages involved in the acquisition of novel vocabulary, achieved at different points during word learning. Indeed, these two stages have been described as *lexical configuration* and *lexical engagement* [65]. Thus, during the early stage of *lexical configuration*, the specific features of the surface word-form are acquired, such as its orthography, phonology or meaning, with a relatively short exposure allowing the fast acquisition of memory traces for novel word-forms. Later on, during the *lexical engagement* stage, the intensive exposure to these stimuli allows its integration into the lexicon, and hence its dynamic interaction—in terms of facilitation and competition—with other word units at similar processing level. Different behavioral studies have reported data supporting this two-stage process [35, 42, 47, 111], showing the early phase of configuration as a necessary condition for novel word learning. Indeed, the foundations of new word acquisition are likely established during this early stage, through the formation of episodic memory representations. Later on, the word’s connections become distributed over the entire language neural network due to extensive experience, going beyond the initial encoding in isolated episodic use. In the present study, we aimed to further investigate the neurophysiological underpinnings of the early lexical configuration in the written domain, that is, the rapid acquisition of novel word´s orthography.

In contrast to a substantial amount of ERP research in spoken language, focused on rapid learning of pure phonological word-forms, the evidence regarding the putative neurophysiological mechanisms underlying the acquisition of orthographic word-forms is rather limited. The vast majority of studies in this strand of research (including the ones listed above) combined training in both the orthography and the meaning of new words [8, 14, 76, 82], whereas only few of them evaluated the brain dynamics underlying purely orthographic learning as such (e.g., [11, 13, 80]). However, the underlying neural mechanisms for the acquisition of a novel surface form *per se* and the meaning attached to it are likely dissociable, with one related to the analysis of visual features and the orthographic recognition of the surface form and the other related to the access of its associated concept [23, 86]. Indeed, ERP studies on visual word recognition have provided evidence of dissociation of orthographic and semantic processes at neurophysiological level. The orthographic processes related to the extraction of visual features and word-form analysis appear to be reflected in early brain responses, elicited within the first 250 ms of word processing [3, 4, 10, 25, 59]. In particular, the amplitude of the P200, a fronto-centrally distributed ERP component, is known to be modulated by the access of the orthographic word-form, with larger responses for high-frequency words in comparison to low-frequency words or to pseudowords^2^, therefore, this ERP is considered to be an index of holistic word-form recognition [7, 21, 67, 89, 114]. On the other hand, the access of word meaning has in turn been found to elicit later brain responses, ~250–500 ms after stimulus onset, most notably reflected in the amplitude of the N400 component, already mentioned above. This parietally-distributed negativity is largely known as a robust brain correlate of lexico-semantic processing, sensitive to both lexical status and semantic context of the stimuli [9, 56], Federmeier and Kutas 2011). Thus, smaller N400 responses are considered to reflect the ease of processing and integrating the word into the preceding context, as well as its context-driven expectancy. Besides N400 semantic priming effects (in which prior presentation of semantically related word reduces the N400 amplitude), ERP research has shown the sensitivity of this component to the physical repetition of the stimuli, with more positive-going (i.e. less negative) responses for repeated than for unrepeated stimuli, which is interpreted as a sign of facilitation in the semantic access because of the repetition-induced pre-activation of lexico-semantic entries [30, 63, 85, 94]. Importantly, although, based on the above, the orthographic and semantic analyses might be considered as consecutive processes, there is also evidence of earlier lexico-semantic activation during visual word recognition (between 100 and 200 ms), suggesting a cascaded-interactive nature of the linguistic processing [31, 32, 46, 91, 97]. This, in turn, implies that the build-up of a surface word-form representation in the presence of semantics may obscure the brain dynamics responsible for the acquisition of orthographic traces for novel words as such. Therefore, to fully understand this process, it seems crucial to determine the activation patterns which occur during the visual encounter with novel written word-forms per se and enable the formation of orthographic traces, without confounding them by semantic effects taking place in parallel.

However, the majority of studies addressing fast learning of novel written word-forms have used a meaning-based training approach—and consequently reported the modulation of the N400—thus preventing us from disentangling putative orthographic surface-level from semantic effects. Some of the very few ERP studies using semantics-free paradigms have shown that the brief exposure to novel written word-forms involves the activation of episodic memory processes [11, 13]. In particular, a short and meaningless training with novel written word-forms (only six exposures) was found to produce an enhancement of the so-called Late Positive Component (LPC), to the point that differences between responses to these stimuli and those to known words disappeared by the end of the task [13]. This ERP component is a late (~500–700 ms) central positivity, typically observed in repetition and old-new paradigms, and its enhancement has been related to the encoding and strengthening of episodic memory traces that enable recognition of previously presented stimuli (see Rugg and Curran [95], for a review).

The visual training carried out in these studies did not produce, however, any effect linked to earlier lexical processes, indicative of orthographic learning (reflected, for instance, in the modulation of P200). Even the well-stablished N400 repetition effect, mentioned above, was either missing or very weak in these studies, despite the repeated exposure to novel stimuli over the training. One plausible explanation could be the use of a non-natural reading context—a lexical decision task—for the training of novel word-forms [11, 13]. Such manipulation could enhance the attention—and hence linked episodic processes—on novel word-forms in order to actively categorize them during the task, masking or blurring the activation at the earlier stages of processing. Indeed, similar LPC enhancements have been also found in other studies in which an explicit categorization (i.e. semantic judgement) was required for stimuli previously trained in both orthography and meaning (e.g.: [6, 8, 82]), which suggests the link between this late modulation and non-lexical processes driven by particular task requirements (explicit attention-demanding lexical or semantic categorization).

In more detail, previous ERP studies have shown the influence that attentional processes, driven by such categorization demands, commence at earlier stages during visual word recognition [38, 57, 60, 93]. In these studies, the N400 effect found under lexical or semantic categorization tasks is actually overlapped by the modulation of the P300, a component related to attentional mechanisms activated to accomplish the task [87, 88], thus confounding the interpretation of ER effects at the lexico-semantic stage of word processing. Furthermore, the modulation of the P300 has been found to differ across tasks varying in the amount of explicit demands over the stimuli [12, 93], with the subsequent P300-N400 overlap observed at high-level (i.e. lexical decision task) but not at low-level demand tasks (i.e. reading task). Therefore, enhanced attention driven by specific categorization of the novel words may prevent the observation of changes at early (most crucially, orthographic) stages of their processing. In this sense, the use of training contexts which do not involve overt categorization or other behaviorally specific responses to the trained stimuli seems essential to study the effects of visual training at early stages of their processing.

Indeed, earlier effects relative to lexical processing of novel word-forms have been found in visual domain when the training involved a more automatic, “task-free” learning [80], in a similar way as previously found in spoken domain [53, 54, 102, 104, 115]. In particular, in their MEG study, Partanen and colleagues found that unattended exposure to novel meaningless written word-forms during a non-linguistic distraction task caused a modulation of the brain response at earlier stages of stimulus processing (around ~100 and 200 ms). No modulation was found at later time windows (around ~300 or 500 ms) in this attention- and task-free non-semantic paradigm. Remarkably, the visual exposure implemented in this study was outside the focus of reader’s attention, using parafoveal tachistoscopic presentation of stimuli, and thus advocating automaticity of the memory trace build-up memory even in visual domain. However, reading—especially involving encounters with novel visual information—is usually an attentive process. Moreover, the training implemented in the Partanen et al. visual study, as well as in similar studies in spoken domain, involved a massive exposure to novel word-forms, with many repetitions over the experimental session (over 100). This approach contrasts with the short exposure carried out in the ERP studies using attentive-categorization tasks for training, and particularly with behavioral studies in this strand of research, wherein training paradigms are usually more similar to the learning conditions in visual domain than in the above M/EEG studies (i.e. attentive low-level demand tasks, such as single-word or sentence reading, in which few exposures of the novel word—usually less than 10—are provided).

In sum, the putative brain mechanisms for the formation of purely visual word-form representations require further investigation. In particular, more studies are needed that could avoid the confound between orthographic learning and semantic or categorization processes, and would employ more natural paradigms similar to those used in behavioral research, involving brief and attentive exposure to new words and using reading, rather than lexical categorization or other unrelated visual tasks. Here, we asked whether a brief training—up to six exposures—with novel word-forms in an attentive reading task (resembling the training conditions in behavioral studies), could produce neural changes indicative of a build-up of lexical memory traces. More specifically, we hypothesized that this training would allow us to detect changes particularly related with the orthographic learning of the novel written word-forms, in the absence of other confounding factors. Therefore, it was expected this learning would be reflected in the modulation of the P200 component, a known neural marker of orthographic word-form access. Besides this, we might also expect modulation of N400 and LPC components, since changes in these ERPs have been often found in previous studies addressing novel word learning. However, since our training paradigm avoids precisely the conditions that are believed to affect these late responses (such as inclusion of semantic context or requirement of stimulus categorization along the task), the predictions for these components are somewhat less straightforward. Nonetheless, since the repeated exposure to novel word-forms was expected to cause the formation of new orthographic traces, their activation could, in turn, facilitate the lexical processing of these stimuli in upcoming encounters, which might be reflected in the progressive reduction of the N400. Moreover, the re-activation of word memory traces through their repeated exposure could potentially trigger the episodic processing for these stimuli, which might be reflected in the LPC enhancement (although, notably, this effect has been particularly linked to categorization demands, which are absent in the present training task). Accordingly, EEG methodology was used to explore changes in both early (150–250 ms) and late (250–800 ms) brain’s electrical signals, generated on-line during the repeated exposure to novel written word-forms in a reading task. The impact of each individual encounter with the novel word-form was tested by means of a single-trial, cluster-based random permutation analysis of EEG data. Thus, rather than just comparing pre and post training effects, by using this fine-grain method we also estimated the contribution of each repetition along the training into the changes in the brain electrical response elicited by novel word-forms. In addition, an exploratory, data-driven analysis of neural source estimation was carried out in order to identify the brain generators responsible for the ERP modulations found at surface level. We hypothesized that, if an early ERP modulation (i.e. P200) actually encodes the putative orthographic learning of the novel word-forms, then the differences in the processing of these stimuli before and after the training would be observed in the brain regions related to orthographic processing (such as left lingual and fusiform gyrus) [78, 83, 84, 87, 90].

## Results

**Fig. 1:**
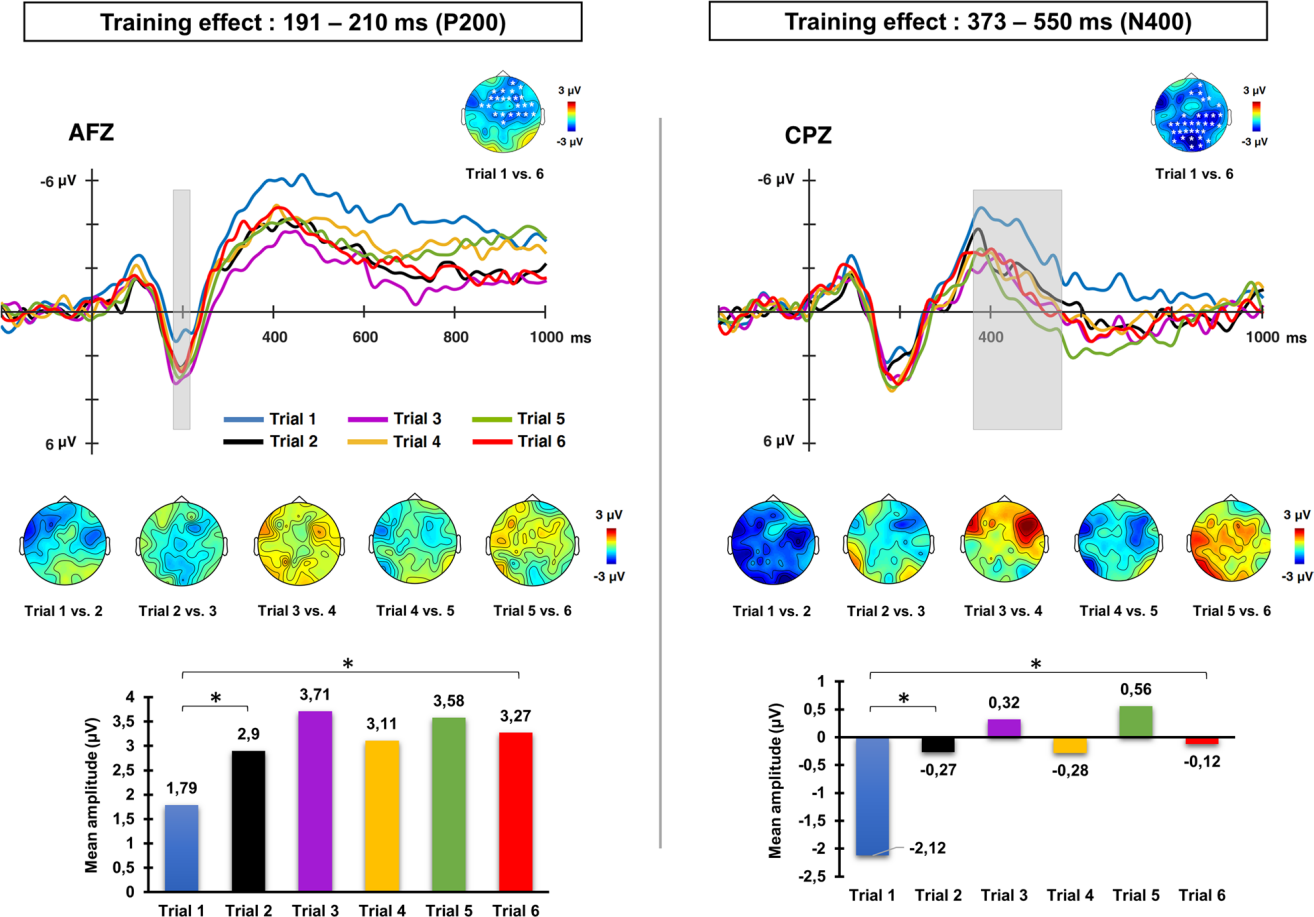
Averaged ERP waveforms at midline scalp sites for novel word-form exposures across the six different training trials. Panels on the left and on the right show the training effects found at P200 and N400 intervals, respectively. Topographic maps above each set of ERP waveforms depict scalp distribution and electrodes in which the general effect of novel word training (first vs. last trial of exposure) was significant in the cluster-based random permutation analysis (time windows are highlighted in grey shaded areas). Topographic maps below each set of waveforms show the scalp distribution of the differences between novel word-forms across each new exposure. Bar graphs below each panel show the mean amplitude of each ERP obtained for novel words across the training blocks. Cluster analysis for each pair-wise comparison carried out across training trials revealed that changes at both P200 and N400 time windows were very fast (already at the second exposure) and stable over the rest of the training

Cluster analysis carried out for the effect of training (contrasting novel word-forms at the beginning and at the end of the training) resulted in two significant clusters of differences, obtained in the tests carried out over the early (150–250 ms) and late (250–800 ms) temporal segments. The first cluster extended from 191–210 ms (*t*(25)= −3.17, *p*= 0.041), with maximal activity at 201 ms, showing a fronto-central distribution and revealing more positive amplitude for novel words presented in the last than in the first block of repetitions (diff. 1st vs. 6th trial= −1.48 µV). The second cluster of differences extended from 373–550 ms (*t*(25)= −3.06, *p*=0.005), maximal at 460 ms, with a centro-posterior distribution showing less negative amplitude at the last than at the first trial (diff. 1st vs. 6th trial = 2.00 µV). Both the latency and the scalp distribution of these two effects likely suggest the modulation of P200 and N400 components, respectively, as can also be observed in the averaged waveforms of ERPs and topographic maps plotted in Fig. 1.

The activity at each resulting time window (191–210 ms and 373–550 ms) was averaged across significant channels and complementary analyses were carried out in order to further explore the effect of each single repetition along the entire orthographic training. Results for P200 time window (191–210 ms) showed significant increase of the positivity elicited by novel word-forms across training trials (see Fig. 1 for mean amplitudes values elicited across exposures). Crucially, the strongest change was found from the 1st to the 2nd training trial (*t*(25) = −2.47, *p* = 0.036; diff. = −1.12 µV) whereas no significant differences were found from the 2rd to the remaining trials of exposure (all *p*s > 0.05). Similarly, the repeated exposure to novel word-forms was found to modulate the N400 amplitude especially in the beginning of the training; thus, the strongest reduction in the N400 amplitude was observed from the 1st to the 2nd trial (*t*(25) = −2.99, *p* = 0.001; diff. = −1.85 µV), whereas no significant modulations were found between subsequent blocks (all *p*s > 0.05, see Fig. 1 for details). Therefore, this pattern of results shows that the modulation in both P200 and N400 time windows was very fast (taking place after the first exposure) and was maintained across the training session.

**Fig. 2:**
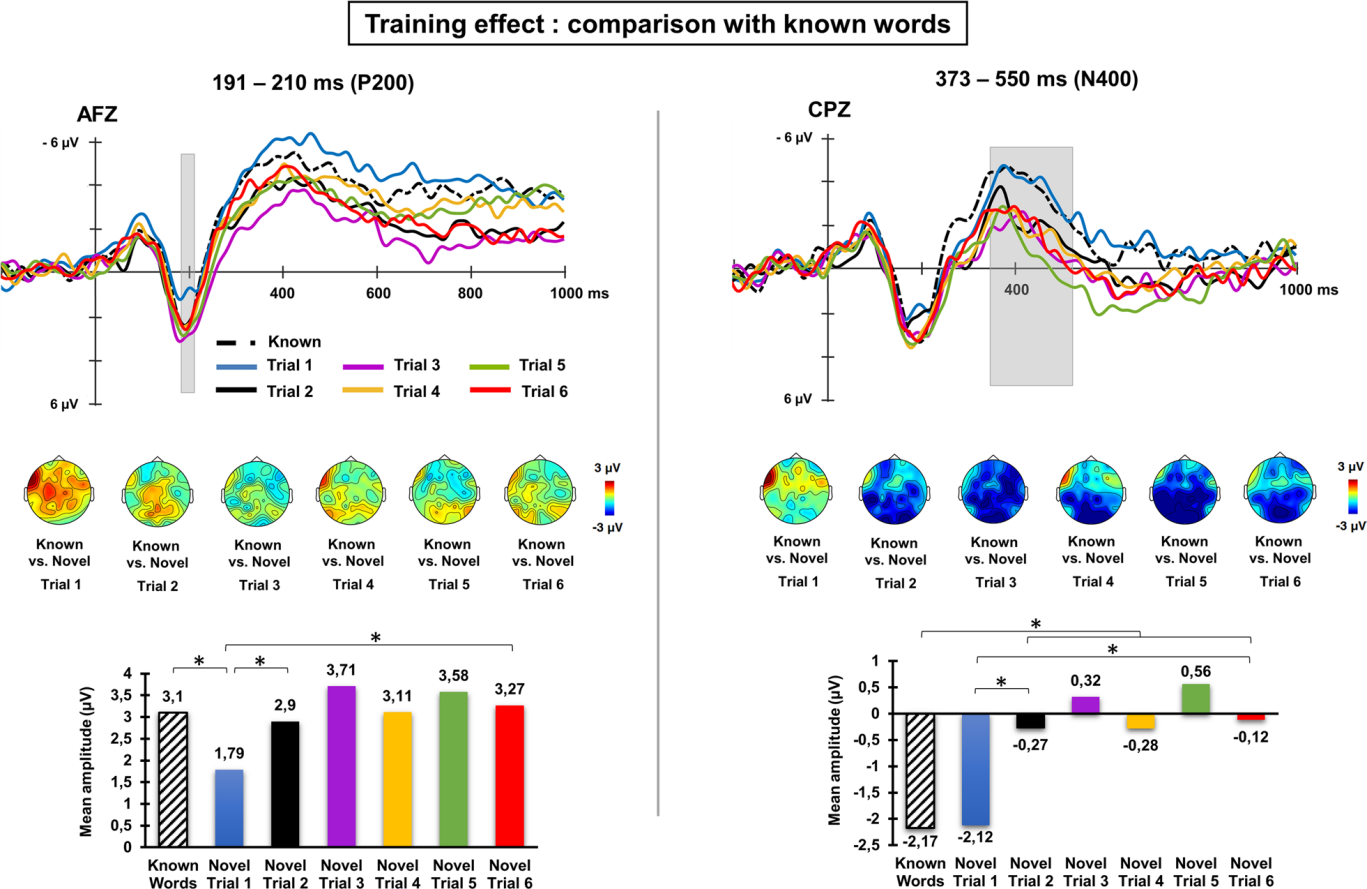
Averaged ERP waveforms for control known words and for novel words across the six different training trials. Panels on the left and on the right show the training effect found in the P200 and N400 time windows, respectively (highlighted in grey shaded areas). Topographical maps below each set of waveforms show differences in scalp distribution between known and novel words across the training trials. Bar graphs below each panel show the mean amplitude of each ERP obtained for known words and for novel words across the training blocks. For the P200 time window, pair-wise comparisons revealed that mean activity elicited by control and novel words differed at the first trial but became similar already at the second exposure of novel words and was maintained throughout the rest of the training. However, for the N400 time window, pair-wise comparisons revealed that lexical differences emerged after the second exposure with novel word-forms and were maintained across the rest of training trials

Additional comparisons with control stimuli—known words—over the averaged time windows and significant channels identified in previous cluster analysis resulted in a significant lexicality effect in the P200 time window, with a stronger P200 response exhibited by known words in comparison to novel word-forms presented at the 1^st^ trial (*t*(25) = 2.84, *p* = 0.017; difference between known words vs. novel word-forms at 1st trial: 1.30 µV). However, with training, these differences vanished, with both novel and known words showing similar brain activity already at the 2nd exposure and until the end of the task (all *p*s > 0.05; see Fig. 2). Therefore, the modulation of the brain’s electrophysiological response produced by the orthographic training of novel word-forms reduced the P200 lexicality effect such that it was eliminated after just one visual repetition. A somewhat different pattern of effects was found for the N400 time window. No significant difference was detected between known and novel word-forms presented at the 1st trial (known words vs. novel word-forms difference at 1st trial: −0.05 µV, *p* > 0.05). However, lexical differences emerged at the second exposure to novel word-forms (*t*(25) = −3.03; *p* = 0.004; diff. = −1.90 µV), which were maintained across the training for all remaining trials (all *ps* < 0.01).

**Fig. 3:**
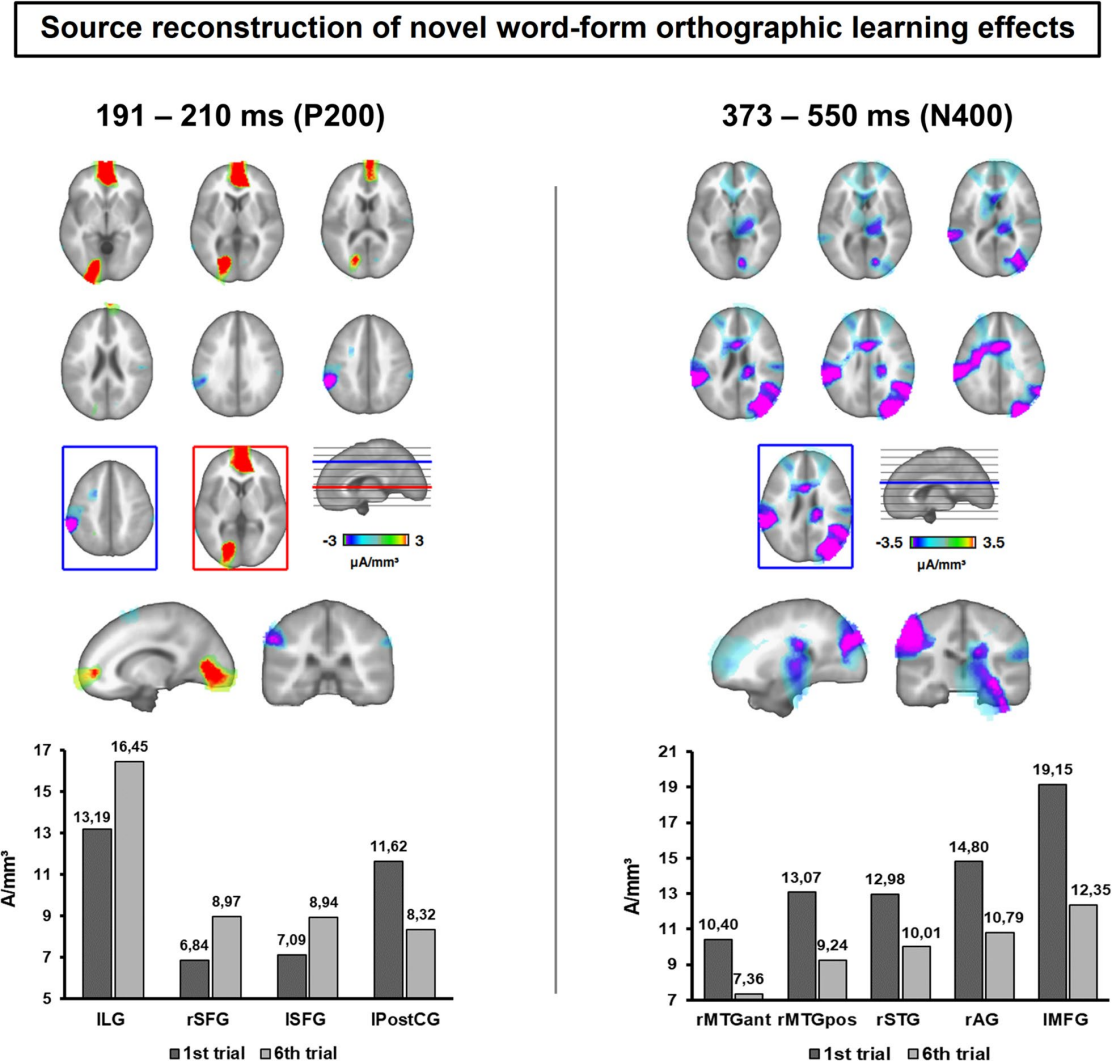
LAURA neural source reconstruction of the ERP training effects (last vs. first exposure with novel word-forms) obtained for P200 and N400 time windows. T-maps represent the brain location of differences in current source density between the last and first exposure to novel words, with the loci of maximal differences framed in red. For the early, P200 time window (left panel), the left lingual gyrus (lLG) and bilateral superior frontal gyrus (SFG) were found as the most probable neural sources for the P200 increase obtained at scalp level, whose activity was found stronger along the exposures with novel word-forms. For the late time window (right panel), the neural generators of the N400 reduction were most expressed in the right middle and superior temporal gyrus, right angular gyrus and the left middle frontal gyrus, whose activity was found reduced from the first to the last exposure with novel written word-forms. Graphs show the mean current source magnitudes at significant ROIs. Labels refer to neural sources: lLG (left Lingual Gyrus), rSFG (right Superior Frontal Gyrus), lSFG (left Superior Frontal Gyrus), lPostCG (left Postcentral Gyrus), rMTGant (right Middle Temporal Gyrus, anterior section), rMTGpos (right Middle Temporal Gyrus, posterior section), rSTG (right Superior Temporal Gyrus), rAG (right Angular Gyrus), lMFG (left Middle Frontal Gyrus)

Neural source reconstruction of the orthographic training ERP effect (novel word-forms presented at first vs. at last trial) was carried our using LAURA distributed source estimation method. Two ROIs were identified as the most likely neural contributors to the early P200 increase observed at surface level, namely the left lingual gyrus (left LG, maximal in x = −16.72, y = −55.47, z = 5.88, Talairach Coordinates, corresponding to BA 18, Talairach and Tournoux [110]) and the bilateral superior frontal gyrus (right SFG: x = 3.34, y = 62.33, z = −0.008; left SFG: x = −3.34, y = 62.33, z = −0.008, corresponding to BA 10). See Fig. 3 (left panel). Further analyses carried out in both ROIs revealed the increase of activation from the first to the last exposure with the novel written word-forms (left LG: *t*(25) = 2.84, *p* = 0.009, diff. 1st vs. trial: −3.27 A/mm^3^; Right SFG: *t*(25) = 2.86, *p* = 0.008, diff. 1st vs. 6th trial: −2.13 A/mm^3^; Left SFG: *t*(25) = 2.84, *p* = 0.009, diff. 1st vs. 6th trial: −1.84 A/mm^3^). Consequently, differences exhibited between novel and known words at the beginning of the training (left LG: *t*(25) =  −2.075, *p* = 0.048, diff. novel vs. known: −3.06 A/mm^3^; Right SFG: *t*(25) = −2.19, *p* = 0.038, diff. novel vs. known: −1.93 A/mm^3^; Left SFG: *t*(25) = −3.24, *p* = 0.003, diff. novel vs. known: −2.56 A/mm^3^) were found as eliminated at the last exposure with novel word-forms (all *t*s(25) < 1, all *p*s > 0.4). In addition, the left postcentral gyrus was also identified as another likely neural source of the training effect (left PostCG, x = −56.85, y = −20.88, z = 41.50), showing the decrease of activity from the first to the last training trial (*t*(25) = −2.62, *p* = 0.015, diff. 1st vs. 6th trial: 3.3 A/mm^3^) and thus causing the increase of differences with control known words (first trial: *t*(25) = −0.43, *p* = 0.66, diff. novel vs. known: −0.46 A/mm^3^; last trial: *t*(25) = −2.85, *p* = 0.009, diff. novel vs. known: −3.76 A/mm^3^).

Figure 3 (right panel) shows the most likely neural sources responsible for the reduction of N400 activity, identified in the right middle and superior temporal gyrus (rMTG, posterior section, x = 36.78, y = −61.09, z = 24.84, corresponding to BA 39; rMTG, anterior section, x = 36.78, y = −3.98, z = −14, corresponding to BA 21; rSTG: x = 36.78, y = −54.85, z = 18.30, corresponding to BA 22), right inferior parietal lobule (including angular gyrus, rAG, x = 30.09, y = − 60.09, z = 31.04, corresponding to BA39/40) and the left middle frontal gyrus (lMFG, x = −43.47, y = 12.14, z = 46.07, corresponding to BA 6). At these locations, a reduction of activity was found from the first to the last exposure to novel word-forms (rMTG, posterior section: *t*(25) = −5.008, *p* = 0.000, diff. 1st vs. 6th trial: 3.84 A/mm^3^; rMTG, anterior section: *t*(25) = −3.22, *p* = 0.004, diff. 1st vs. 6th trial: 3.03 A/mm^3^; rSTG: *t*(25) = −3.73, *p* = 0.001, diff. 1st vs. 6th trial: 2.97 A/mm^3^; rAG: *t*(25) = −4.11, *p* = 0.000, diff. 1st vs. 6th trial: 4.01 A/mm^3^; lMFG: *t*(25) = −4.63, *p* = 0.000, diff. 1st vs. 6th trial: 6.81 A/mm^3^) thus increasing differences between novel and known words from the beginning (all *t*s < 1.6, *p*s > 0.1) to the end of the training (rMTG, posterior section: *t*(25) = −3.57, *p* = 0.001, diff. novel vs. known: −4.78 A/mm^3^; rMTG, anterior section: *t*(25) = −4.36, *p* = 0.000, diff. novel vs. known: −4.54 A/mm^3^; rSTG: *t*(25) = −2.79, *p* = 0.01, diff. novel vs. known: −3.58 A/mm^3^; rAG: *t*(25) = −2.54, *p* = 0.018, diff. novel vs. known: −4.27 A/mm^3^; lMFG: *t*(25) = −4.11, *p* = 0.000, diff. novel vs. known: −9.53 A/mm^3^).

## Discussion

In this study we report ultra-rapid changes in the brain’s electrophysiological signal elicited by novel meaningless written word-forms, showing the influence of a very short training (6 exposures only) at both early and late lexical stages of the processing of these stimuli. In particular, the single-trial analysis carried out in this study revealed that the strongest change in the brain electrical response to novel word-forms took place between their first two exposures, reflected in the modulation of both P200 and N400 components.

The brief orthographic training with novel word-forms produced a strikingly fast and stable enhancement of an early positivity, as observed in the amplitude of the P200 component. This ERP component has been related to the extraction of orthographic and phonological word features at early stages of word processing [7], Carreriras et al. 2005; [67, 89, 114]. More specifically, smaller P200 amplitudes have been associated with more sub-lexical orthographic activation. In this sense, the P200 enhancement could be related to a modification of sub-lexical orthographic process, switching from the letter-by-letter decoding to a more holistic lexical-type access of newly formed representations. This interpretation is also supported by the elimination of P200 differences between trained and already known words, possibly reflecting the process of establishing the whole-word recognition strategy for these new items, similar to that used for the reading of well-known lexical stimuli. Note that our known and novel stimuli were matched in various low-level psycholinguistic features (incl. syllabic and bigram frequency), which implies that this dynamic likely reflects whole-form acquisition rather than a letter- or bigram-related effect. Furthermore, the findings at source level are also in agreement with this argument, with the left lingual gyrus as one of the most likely neural sources responsible for the P200 enhancement found at surface level. Indeed, this visual region has been proposed, together with the fusiform gyrus, as part of the word-form processing system involved in the orthographic analysis of real versus false fonts or non-letter strings, carried out during early stages of reading (Nobre et al. 1994, Petersen et al. 1988, 1990; Puce et al. 1996). Whereas the left fusiform has been related to the processing of local features, the left lingual gyrus is engaged in global shape processing, activated when attention is directed to the processing of global parts, such as the whole word-form [39, 40, 75]. Thus, the increase of activation found in this region likely indicates the stronger whole-shape discrimination for the novel written word-forms through their repetitions. Indeed, whereas novel words initially exhibited lower activation than known words at this region, as was similarly reported in previous studies [43, 74], novel words reached a similar level of activation than known words after the training. Taken together, these results likely indicate the enhancement of a whole-form based reading strategy for novel written words as a consequence of this short visual exposure.

These findings are in line with cognitive models developed in psycholinguistics to account for reading processes, and particularly for the visual recognition of already known and newly-experienced words [23, 86]. According to these models, the more often a particular form is encountered, the lower is the threshold for its activation in the orthographic lexicon and therefore, the faster its visual recognition is. Thus, repeated visual exposure with novel word-forms allows the reader to pass from a sub-lexical reading, which operates by means of a serial phonological decoding of each grapheme into its corresponding phoneme, to a holistic reading, characterized by parallel letter decoding. Thus, the P200 modulation found in the present study along the visual exposure to novel written word-forms, as well as the estimated neural generators of this effect, likely reflect neurophysiological changes that underlie the evolution from a sub-lexical to a more lexical, whole-word reading strategy.

On a more cautious side, it may in principle be possible that early ERP modulations found across repetitive stimulus presentation are not language or learning-specific and simply reflect unspecific sensory-level effects of stimulus repetition. However, this non-linguistic explanation does not seem likely, given that repetition effects are typically expressed as suppression/habituation of ERP responses, which is not what we observe here [48, 77], although also see [112] for increased neural responses during repetition). The present changes in P200 amplitude do not show a suppression through the training, but instead manifest a clear facilitation, as predicted by the theoretical account of new memory trace build-up and activation. Moreover, as a result of training, P200 responses to novel written word-forms became similar to those elicited by known, already lexicalized words as a consequence of their repeated exposure, which also speaks to the linguistic nature of this activity pattern. Nonetheless, to fully validate this explanation and rule out the habituation vs. language-related nature of the effects, future experiments should use additional control conditions including familiar words and non-orthographic visual patterns as stimuli (i.e. symbol strings),indeed, the repetitive presentation of non-orthographic stimuli together with the set of main experimental word-forms could help disentangle orthographic from perceptual learning effects while avoiding potential confounds introduced by the repetition of meaningful stimuli (such as formation of new semantic associations, similar enhancement of orthographic memory traces for both sets, etc.).

A similarly fast effect of visual repetition was reflected in the amplitude of an N400 response, showing a remarkable decrease from the first to the second visual presentation of the novel written words, which also remained stable until the end of the training. As reported in previous ERP studies with novel written words trained under meaningful contexts [8, 14, 76, 82], such a reduction in the N400 time interval could reflect the facilitation in the lexico-semantic access of novel stimuli, due to preactivation of the respective concept, previously associated through repetition. However, taking into account that in the present training context we only deal with visual word-forms devoid of semantic content, such an N400 effect cannot be generated by semantic activations per se. In fact, given the novel word-forms trained in the present study were unique stimuli, not derived from real words, such an N400 modulation could not be triggered by accessing the meaning of any related word either. Other explanations, therefore, must be considered for the N400 modulation.

Importantly, the repetition of linguistic stimuli is considered to produce the formation of memory representations, which contain recently processed information whose pre-activation facilitates the processing of the repeated stimuli at each new encounter [106]. Such facilitation, understood as an easier or more fluent processing, is reflected in the present study already at the early stage of the linguistic processing. Thus, the activation of such new mental representations, containing orthographic, surface-related information, contributes to the enhancement of a whole-form processing strategy for these stimuli, as indexed in the P200 modulation. However, even if these mental representations lack meaning, the knowledge gained through the exposures likely contributes to the ease or their processing at a later stage, as reflected in more positive-going N400 responses, typically associated with less effortful lexico-semantic processing [9, 55, 56]. Indeed, previous studies have reported similar N400 reduction effects caused by repetition of meaningless stimuli, including novel stimuli not derived from real words [30, 63], they interpreted this effect as driven by the intrinsic nature of textual stimuli, which suggests all orthographic stimuli as potentially meaningful and thus activates language-based processes during their silent reading, including orthography (P200) and lexical semantics (N400). That could in principle be true for the novel word-forms trained in the present study, since these stimuli were in fact real—but extremely infrequent and hence unknown—word-forms with a specific meaning attached to them, thus, the semantic nature of these stimuli could theoretically boost the orthographic learning and contribute to the rapid ERP changes observed, although this effect is improbable taking into account these stimuli were completely unknown to our participants. Future comparisons across different stimuli sets could answer the question whether the ecological value of real linguistic stimuli (in comparison to artificially created ones) underlies the boost of rapid word learning observed in ERP effects. Nonetheless, from that obligatory-semantics view discussed in aforementioned studies, and in the context of a learning task in which these stimuli were intended to be attended and learnt as much as possible, the N400 reduction observed here likely reflects the increased ease of their lexical processing, caused by pre-activation of previously repeated information containing surface whole-form rather than conceptual features. Indeed, the increase in N400 differences between control stimuli and novel words also suggests the activation of such facilitatory memories for repeated stimuli, in comparison to non-repeated control words.

Data from neural source estimation is also in agreement with the view of the N400 reduction as a language-related effect, connected to the potential lexico-semantic status of the stimuli; the localization of this effect revealed a set of areas typically associated with the lexico-semantic processing as the most likely neural generators of this ERP modulation, namely the right middle and superior temporal gyri as well as the right inferior parietal cortex (including the angular gyrus). These findings are in agreement with previous literature, which has reported these language-related areas among those responsible for the N400 response [36, 52], see [64], for a review). Moreover, the specific pattern of N400 source activation obtained in the present study, showing the decrease of brain activity at temporal regions with novel word repetition, corroborates a number of previous studies [69, 98, 99]. Using similar stimulus repetition paradigms to the one employed here, with no addition of semantic information, these studies found the decrease in the left temporal and frontal gyri as neural generators of the N400 reduction obtained at scalp level across word repetitions, this effect is considered to reflect the decrease of unnecessary or redundant activation, due to the pre-activation of the word representation after its previous presentation. Importantly, the posterior section of the middle temporal gyrus and surrounding areas including superior temporal sulcus and inferior parietal gyrus, found here as the main generators of the N400 modulation, have been proposed as the best candidates for the storage and access of lexical, rather than semantic representations [34, 49, 64]. This supports the argument that the present N400 modulation likely reflects lexical facilitation caused by pre-activation of whole-form surface representations for previously presented stimuli. Nonetheless, the right-hemispheric activation found in the present study does not follow the left lateralization typically observed for language, however, it must be noted that the right-hemisphere distribution of the N400 responses and its associated neural sources has been also reported in the literature, proving the right hemisphere as lesser but robust generator of this ERP [24, 52, 58, 113]. Indeed, a recent N400 study has reported a very similar pattern of source activation as observed here, with the activity decrease in the right superior and middle temporal gyrus along with stimulus repetition [108], nonetheless, the comparison between both studies must be careful, since the N400 effect reported in Ströberg et al. was found under a semantic priming paradigm, and particularly for familiar words preceded by primes presented repeatedly, hence not directly measured for repeated stimuli as in the present study.

In general, it seems feasible to conclude that the pattern of ERP results found in this study reflects the fast built-up of memory traces during the early stage of word learning. However, despite this fast and sustained memory formation process, it may be difficult to claim that visual word-form representations built for these stimuli have been fully integrated into the mental lexicon at this initial stage. Results found here, particularly the P200 modulation, reflect the fast acquisition of orthographic features for novel trained words, enabling the construction of surface-only word-forms and contributing to their *lexical configuration*. Nonetheless, the lexicalization process for these stimuli is likely still in progress, and a more intensive training and/or consolidation are most probably required for their integration and further *engagement* into the mental lexicon, as suggested in previous studies [6, 17, 27, 33, 42, 72, 73, 107].

In general, the present results are in agreement with previous ERP data, confirming the high speed with which neurophysiological traces for novel written words are built up [8, 14, 76, 82]. Importantly, these previous studies were not able to disentangle orthographic and semantic processes during the acquisition of novel word-forms, as they employed novel stimuli with meanings attached to them. In contrast, the brain dynamics reported here more likely reflect the neural mechanisms underlying purely orthographic processes during the initial acquisition of novel written word-forms, in the absence of semantics. Similar suggestion of fast acquision of purely orthography-based word-forms have been made previously [11, 13, 80]. However, in those studies the attention was not specifically focused on the learning of novel word-forms but was instead diverted to accomplishing other visual tasks, such as the categorization of non-related stimuli during the parafoveal repetition of the novel words [80] or the lexical categorization of stimulus items [11, 13]. Importantly, when the context of training prioritizes the categorization of the trained stimulus instead of their simple visual recognition as in the above mentioned studies, the effect of training is only reflected in the LPC component, a late modulation typically related to episodic memory process. Such processes, probably recruited to carry out the required overt reaction, may distort effects at earlier lexical processing stages and thus confound the effects of learning, as already suggested in previous research comparing effects of novel word learning in high and low demanding tasks [12].

In contrast, here we use a more natural context of training, characterized by the attentive encounter with novel word-forms in a silent reading task, and involving a small number of exposures. This approach allowed us to detect fast orthographic learning effects at both early and late stages in the lexical processing of novel word-forms. Thus, when word learning is carried out under a relatively automatic and free-demand task, the effect of training is only shaping their lexical processing at early and late stages—as reflected in both P200 and N400 modulations, with no effects on later components such as the LPC, linked to episodic, categorization-related processes. Remarkably, the early effect found in the present study is consistent with previous findings both in the auditory [53, 54, 80, 102, 104, 115] and, more recently, in the visual domain [80], during the exposure to novel words under relatively non-attentive conditions of exposure to novel spoken and written word-forms.

## Conclusions

Overall, the present study provides new evidence for rapid word learning in visual domain, even in the absence of a semantic reference. The online neural changes obtained through a very short naturalistic encounter with meaningless word-forms show for the first time the activation of early and late lexical stages of the processing for these stimuli, reflected in the modulation of P200 and N400 components. Therefore, the present data show the remarkable speed of the human brain to evolve from a serial to a whole-form reading strategy—after just a couple of exposures to the novel orthographic stimulus, an ability likely fundamental for learning to read as well as for acquiring new vocabulary when reading. Moreover, these results suggest the impact of the automaticity of the training in obtaining a clear neurophysiological modulation at the early stages of the processing, thus indicating the importance of using low-level demand tasks to study novel word learning. Nevertheless, further research is needed that could overcome the limitations of the present study, providing behavioral measures of learning as well as including the repetition of different types of stimuli as additional control conditions. That would confirm the pattern of rapid word learning obtained at neural level and strengthen the interpretation of the effects found as language-related, indicative of the fast orthographic learning of novel written words. Besides this, future research could extend the present findings by addressing the neural underpinnings of the two stages of the lexicalization process, exploring the conditions that could enable the fast engagement of the novel written words into the reader’s lexicon. In this sense, future ERP investigation might consider the use of post-learning, low-level demand tasks to study the putative interaction of the novel word-forms with other existing lexical entrances after short training periods.

## Methods

Twenty-six students (18 females and 8 males; age range 18–29 years; SD = 2.84) took part in the experiment for course credits. All of them were right-handed, native Spanish speakers with no psychiatric or neurological disorders. Their brain activity was recorded by means of 64 Ag/AgCl active electrodes connected to an EEG amplifier (ActiChAmp, Brain Products GmbH, Gilching, Germany) during a silent reading task. Ocular activity was recorded using horizontal and vertical EOG recordings. During recordings, all electrodes were referenced to the vertex (Cz); two additional electrodes were placed on the mastoid bones for off-line re-referencing of the signal using the mean activity in these two electrodes. EEG signal was amplified and digitized at a 1000 Hz sampling rate and high and low pass filters at 0.1 and 100 Hz, respectively, as well as a 50 Hz notch filter, were applied.

**Fig. 4:**
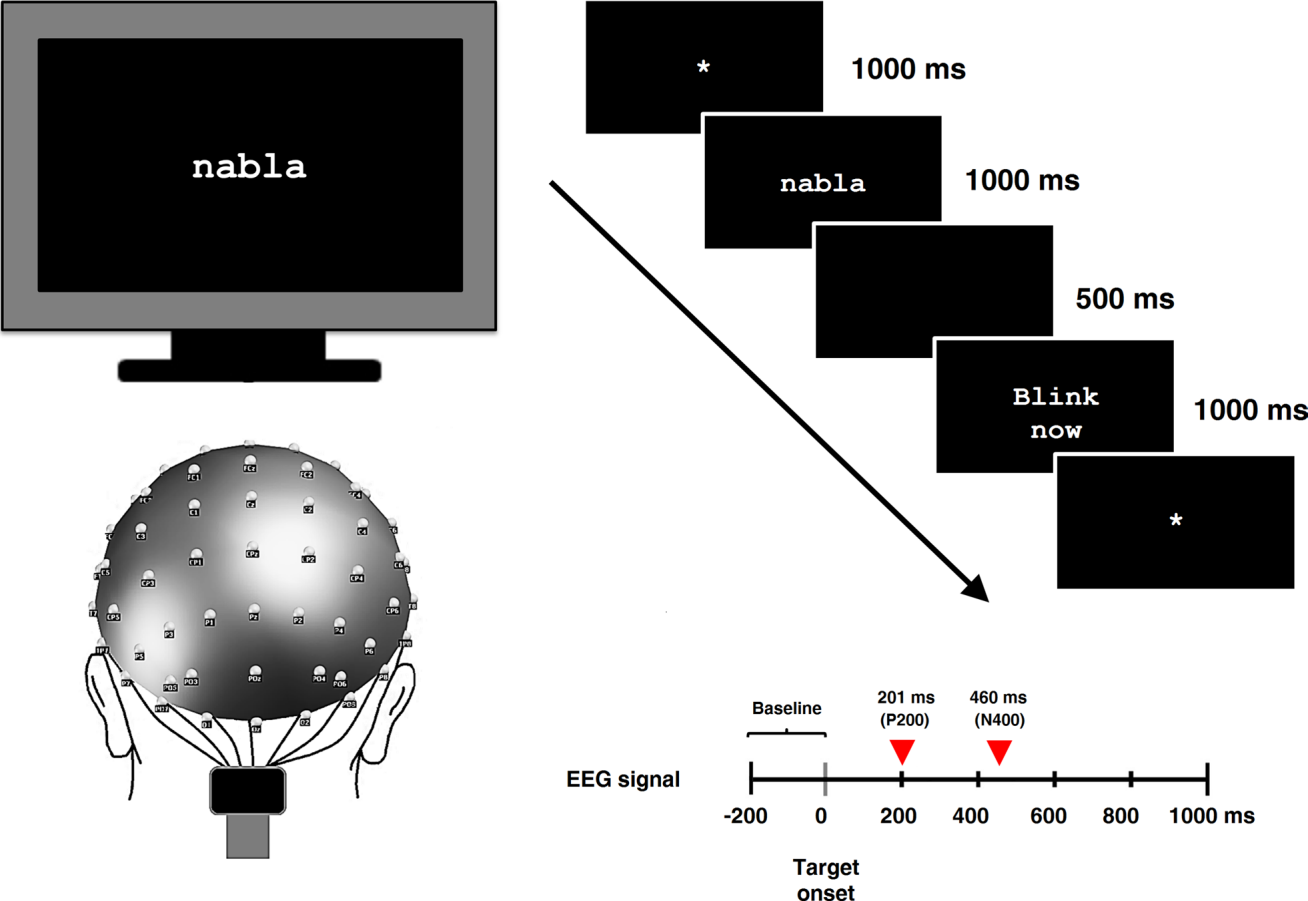
Experimental procedure and sequence of stimuli presentation. During EEG recordings, participants were asked to pay attention to the center of the screen and read silently the stimuli presented. A set of novel written word-forms (e.g.: *nabla*) was presented repeatedly six times across six successive training trials. Additionally, another set of real Spanish known words (e.g.: *nieve*) was presented to participants in order to establish a control comparison between known and novel written words. For both sets of stimuli, equal sequence of presentation was followed with the same elements, with presentation durations indicated to the right of each rectangle. Red triangles on the schematic ERP epoch (lower right) indicate, for each ERP effect, the latencies when maximal ERP changes were found as consequence of the repeated exposure to novel written word-forms

Figure 4 shows the experimental procedure. The reading task included 24 known words (medium frequency Spanish words, extracted from Alameda and Cuetos [1]), used as control items and 24 previously unknown word-forms (obscure words, with mean lexical frequency of 0 occurrences per million, Martinez and Garcia [71]) acting as novel words to be trained. Obscure (or rare) words are real words existing in the dictionary, but due to their very low lexical frequency these stimuli are unknown for participants, thus acting as novel words to be learned. The selection of such stimuli as novel words, instead of building them by changing letters of real words, has been often carried out for the study of novel word learning [2, 41, 82]. This procedure ensures ecological learning effects by means of fully naturalistic materials—new entrances in participant’s native orthography, as well as prevents the activation of real words led by excessive orthographic similarity. Participants were asked at the end of the training, to ensure they were naïve and had no previous knowledge about the novel stimuli. Both known and novel words were disyllabic stimuli, 5–6 letter long (see Table 1 for characteristics of the stimuli). The novel and known words were matched for the number of letters and syllables, mean syllable frequency, bigram frequency and number of orthographic neighbors (independent-samples *t*-tests confirmed no statistical differences, with all contrasts at *p *>.05) by means of the Buscapalabras database [28]. The set of known words (e.g., *nieve,* Eng. snow, *balcón, Eng. balcony*) was presented first, followed by the set of unknown written word-forms (e.g., *nabla,* ancient musical instrument,*jínjol,* a type of buckthorn), which was repeatedly presented in six different blocks, each containing one trial of each stimulus in different randomized order across blocks (hence, each stimulus was exposed across 6 different trials). The presentation of known words was included in order to establish an additional control comparison between already known and novel written words. As a note, the explicit repetition of these stimuli was avoided in order to prevent semantic association between these and novel words, thus ensuring the assessment of purely orthographic mechanisms during novel word learning. Such procedure was also aimed to limit a possible re-activation and strengthening of orthographic traces for known words, a mechanism that could not easily be disentangled from—as well as confounded with—the acquisition of novel orthographic information. The task was introduced to participants as an experiment for learning new words, they were instructed to read the stimuli presented on the screen silently, by means of covert articulation, paying as much attention as possible and trying to learn them. Familiarization trials (using other stimuli) were provided before the start of the task; breaks were taken after each block in order to avoid fatigue. Stimuli were displayed in the center of a computer screen in white, 18-point bold Courier New font over a black background by means of E-Prime 2.0 software (Psychology Software Tools Inc., Pittsburgh, USA). First, a fixation mark was displayed during 1000 ms, followed by the presentation of the stimulus for another 1000 ms. A blank screen was then presented for 500 ms and finally the instruction ‘‘blink now’’ for 1000 ms.

**Table 1.** Main psycholinguistic properties of the stimuli used

	Novel Words	Known Words	*t* (46) value	*p* value
Lexical frequency	0	57.78 (103.99)	–	–
Number of syllables	2 (0)	2 (0)	0	1
Number of letters	5.50 (0.51)	5.50 (0.51)	0	1
Number of orthographic Neighbors	1.42 (1.31)	1.46 (1.21)	− 0.11	0.91
Bigram frequency (token type)	518.92 (285.91)	601.7 (350.51)	− 0.89	0.37
Mean (1st and 2nd) Syllable Frequency	2046.83 (3150.97)	2108.54 (2997.74)	− 0.07	0.94

Stimuli were maximally matched between the experimental conditions. Standard deviation is shown in brackets. Independent-samples *t*-tests confirmed no differences between novel and known words across the variables

Novel words used in the study: cofín, dorna, fudre, bruño, gelfe, nabla, notro, pajel, paila, sisón, cuatí, facón, dolmán, puntel, reitre, roblón, runcho, seisén, holmio, trujal, jínjol, pambil, timple, carmes; Known words: color, toldo, valle, traje, golfo, bicho, litro, papel, nieve, mujer, baile, gafas, balcón, doctor, huella, millón, rastro, violín, garfio, crimen, cactus, césped, templo, pintor.

Preprocessing of the EEG data was carried out using Fieldtrip Toolbox [79]. Raw data were low pass-filtered at 30 Hz and downsampled to 256 Hz. Recordings were epoched between −200 to 1000 ms post stimulus onset and the baseline was corrected using the 200-ms interval preceding the stimulus onset. Independent component analysis (ICA) was used to remove ocular artifacts and a triangular interpolation of bad channels was applied. Additional artifact rejection (using exclusion criteria at ± 100 µV) was applied to remove any remaining contaminated epochs. Data were re-referenced offline to average mastoid reference. Finally, EEG epochs were averaged per subject and per condition and ERPs were computed for novel word-forms at each task block, as well as for known words (with a mean of 20 epochs included per condition). The resulting ERPs were submitted to a cluster-based random permutation analysis in order to test the effect of the orthographic training. This is a method which deals with multiple comparisons in space and time, over the whole ERP segment, and finds clusters (data points in close temporal and spatial proximity) of significant differences between conditions, while effectively controlling for type 1 error [70]. Two steps were followed for cluster-based analyses:

First, the general effect of the orthographic training was studied by analyzing the differences between novel written word-forms presented at the beginning (first trial) and at the end of the task (sixth trial). In particular, two temporal windows were defined, from 150–250 ms and from 250–800 ms, in order to test the training effect at early and late stages of the processing; these two time windows were selected based on previous ERP literature in which early (before 250 ms) and late responses have been distinguished during visual word recognition (see for instance, [7, 21, 50, 55]. Then, the two conditions (novel words in blocks 1 and 6) were contrasted by *t*-tests computed for every sample point across each temporal window (across 1500 sample points for the early temporal window of 150 to 250 ms segment, i.e. 25 time samples × 60 channels, and across 8460 sample points for the late temporal window of 250 to 800 ms segment, i.e. 141 time samples × 60 channels). Those samples below or equal to a predetermined alpha level (0.05) were grouped together based on spatial and temporal adjacency (a minimum of 2 adjacent sample points was required). The cluster effect size was then calculated by taking the sum of all individual *t*-test values of every temporo-spatial grouping (or cluster). In order to correct for multiple comparisons carried out, a cluster-based test statistic method was then implemented. In particular, the null distribution of cluster-level statistic was calculated by randomly assigning ERP segments to the experimental condition (here, 1000 times). A new cluster effect size was calculated after each randomization and the cluster with the largest effect size entered in the distribution. Finally, the cluster was considered significant if the probability of the null hypothesis was below or equal 5%, i.e., if the proportion of cases, in which the values of this distribution were larger than the observed cluster-level statistic. Once a cluster was detected in either of the ERP segments, a new contrast was carried out to further explore the scalp localization of the resulting cluster, averaging its time interval.

Next, in a second step we aimed to study the effect of each single repetition along the whole orthographic training, by using a more detailed, trial-by-trial approach. Thus, for each resulting cluster, a new analysis taking the mean amplitude over the time windows and electrodes of the resulting significant clusters was carried out to contrast novel words presented at specific training trials (i.e., first vs. second, second vs. third, etc.); additional comparisons were also carried out between novel and control known words in each trial. The same cluster-based test statistic method as implemented in the first step was used to account for multiple comparisons during this second analysis.

Finally, brain sources underlying the ERP effect of orthographic training were estimated using the LAURA distributed source estimation method [29], implemented in Cartool Software [18]. The solution space was calculated using a realistic head model, including 4011 nodes defined at regular distances within the grey matter of a standard magnetic resonance image (MRI) template, which is based on the average of 305 healthy adult brain MRIs (created by the Montreal Neurological Institute, MNI, see Evans et al. [37]). Current source magnitudes (ampere per squared millimeter) at each node were calculated for each participant and condition (novel written words at first and last training trial, and control words) over averaged time windows showing significant repetition effects at surface level. The analysis at source level was carried out following a data-driven approach in two steps. First, the resulting density magnitudes for novel words at the first and last trial were contrasted by means of *t*-tests, and source maps for the effect of training were estimated. Then, in a second step, regions of interest (ROIs) were created for those source maps showing largest differences (*t* values >2.5 for at least 10 nearby solution points). The mean values of current source density were extracted for each condition on selected ROIs and submitted to paired *t*-tests for the effect of orthographic training (first vs. last exposure) as well as for the contrast between control and novel words at the beginning and at end of the training.

## Data Availability

The datasets used and analyzed during the current study are available from the corresponding author on reasonable request.

## Footnotes

1 By novel words we understand linguistic stimuli never experienced before and hence unknown, with no information stored about either their auditory/visual form or meaning yet.

2 A *pseudoword* is a word-like sequence of phonemes/letters, observing the phonotactic and orthotactic rules of participant’s language, but devoid of meaning.
